# Otitis media with effusion and atopy: is there a causal relationship?

**DOI:** 10.1186/s40413-017-0168-x

**Published:** 2017-11-14

**Authors:** Mario E. Zernotti, Ruby Pawankar, Ignacio Ansotegui, Hector Badellino, Juan Sebastian Croce, Elham Hossny, Motohiro Ebisawa, Nelson Rosario, Mario Sanchez Borges, Yuan Zhang, Luo Zhang

**Affiliations:** 1 Department of Otolaryngology, Catholic University of Córdoba, Córdoba, Argentina; 2 Department of Pediatrics, Nippon Medical School, Tokyo, Japan; 3 Department of Allergy and Immunology, Hospital Quirón Bizkaia, Erandio, Spain; 4 Department of Pediatric Respiratory Medicine, Regional Eastern Clinic, San Francisco, Córdoba, Argentina; 5 CIMER, Catholic University of Córdoba and Croce Institute, Córdoba, Argentina; 6 Pediatric Allergy Unit, Children’s Hospital, Ain Shams University, Cairo, Egypt; 7 Department of Pediatrics, National Sagamihara Hospital, Sagamihara-shi, Kanagawa Japan; 8 Federal University of Parana, Curitiba, Brazil; 9 Allergy and Clinical Immunology Department, Centro Médico Docente La Trinidad, Caracas, Venezuela; 10 Department of Otolaryngology – Head and Neck Surgery, Department of Allergy, Beijing TongRen Hospital, Capital Medical University, Beijing Key Laboratory of Nasal Diseases, Beijing Institute of Otolaryngology, Beijing, China

**Keywords:** OME, Risk factors, Infancy, Inflammation; eosinophilic otititis media, Allergy, Rhinosinusitis

## Abstract

Otitis Media with Effusion (OME) is an inflammatory condition of the middle ear cleft, acute or chronic, with collection of fluid in the middle ear with an intact tympanic membrane. It is a very common disease in childhood, the most frequent cause of hearing loss in childhood and often requiring surgery. OME is called *chronic* when the fluid in the middle ear persists for more than three months or when the episodes recur six or more times in one year. The current article covers various aspects of OME including definition, epidemiology. Pathomechanisms, risk factors, role of allergy in OME, impact of upper airway disease on OME, eosinophilic otitis media and management of OME.

## Background

OME is the most common disease of the ear in childhood and the most common cause of hearing loss in childhood. Eighty percent of all children have had an episode of this disease by the age of 10 years, mostly by the age of 3 years. The prevalence is about 20% at the age of 2 years with a decreases in prevelance to 8% at the age of 8 years. More than half of these cases are preceded by an acute otititis media (AOM). Dysfunction of the Eustachian tube plays a key role in the development of OME. OME is considered to be a multifactorial disease, which clinically appears on different levels, caused by predisposing factors. Sequence of birth and gender are predisposing factors. Younger siblings as well as children visiting nursery school and smoking by the mother are among the various risk factors. The role of allergic disease in the pathogenesis of OME has been investigated intensively in the last years. There is a broad association from 5 to 80% in childhood.

## Definition of otitis media with effusion

Otitis Media with Effusion (OME), also known as “Glue Ear” or “Secretory Otitis Media”, is an inflammatory condition of the middle ear cleft, acute or chronic, with collection of non-purulent fluid behind an intact tympanic membrane. It is the most frequent cause of hearing loss in childhood and the most common reason for surgery [1, 2]. OME is called *chronic* when the fluid persists for more than three months or when the episodes recur six or more times in twelve months [1, 3].

## Epidemiology

Epidemiological data about OME are controversial and disparate. This disease, which especially affects children, shows a prevalence of 0.6% among adults, in contrast to the fact that 90% of children under two years have had at least one episode and about 80% of preschool children experience OME.

Prevalence of OME has a seasonal maximum in winter and a minimum in summer [4].

## Predisposing factors

The etiology of OME is multi-factorial; but immature function of the immune system and dysfunction of the Eustachian tube are the most important etiologic factors [5]. Many predisposing factors have been identified in patients with chronic OME (Table 1). Age is one of the most important [4]. It has a relatively low prevalence during the first weeks of life, starts to increase around 10 months of age and reaches a peak between ages 2 and 5 years [5]. Conditions and situations that also play a role in the etiology of this pediatric disease include: upper airway infections; bacterial and viral middle ear infections; primary ciliary dyskinesia; day-care attendance and older siblings; very low birth-weight preterm infants; male sex; tobacco smoke exposure (mainly in association with atopy); chronic rhinosinusitis; adenoid hypertrophy (causing mechanical obstruction in the nasopharynx and an inflammatory environmental); and craniofacial abnormalities (e.g. cleft palate) [4, 5]. In young children (under 3 years of age), infective rhinitis is thought to be the most common predisposing cause of OME [1].

**Table 1:** Predisposing factors of Otitis Media with Effusion (OME)

- Age	
- Male Sex	
- Craniofacial abnormalities (e.g. cleft palate)	
- Day-care school and attendance	
- Adenoid hypertrophy	
- Atopy	
- Tobacco smoke exposure	
- Upper airway infection (acute otitis, chronic rhinosinusitis, infective rhinitis)	
- Extreme pre-term	

The role of allergic disease in the pathogenesis of OME has been investigated intensively in recent years. An allergy-triggered cascade is supposed to cause obstruction of the Eustachian tube, which again could lead to an OME [6–9]. In a cohort study, OME was associated with concomitant allergic rhinitis (OR = 2.29, CI = 0.97–5.39, *P* = 0.058) but not with non-allergic rhinitis, asymptomatic sensitization, asthma or eczema [10]. But this association seems to be significant in children 6 years of age and older; whereas there is no significant association in younger children [8].

## Signs and symptoms and diagnosis

Patients with otitis media with effusion present different grades of conductive hearing loss according to the type of fluid or effusion (serous or mucous) [11]. Autophony and tinnitus are usual as symptoms. Hearing loss can range from 15 to 40 dB [12]. These symptoms, (without pain) do not cause children to complain because they are a silent process. Some levels of distraction or poor school performance are indirect signs to consider [13]. Symptoms are often mild or minimal. They can vary based on a child’s age.

Initially the effusion is serous as a transudate. Then, due to the histological changes of the middle ear mucosa (by an increase of goblet cell metaplasia and mucus glands), the liquid becomes seromucous, then mucoid and finally, the effusion becomes thick and stringy, like a glue (gummy ear). On the tympanic membrane which is originally intact, some trophic changes appear and the presence of atelectasis due to negative pressure and subsequent retraction (pockets) is common in chronic processes [12]. These are the sources of more complicated diseases such as chronic otitis media or cholesteatoma.

Apart from the classic clinical features mentioned above, in 1984 Tomioka [14] described a new clinical pattern characterized by high resistance to treatment associated with asthma and nasal polyposis. These patients have a marked viscosity, and this effusion is characterized by high numbers of eosinophils. This special presentation of otitis media with effusion is called *eosinophilic otitis media*. Another feature is that these patients have a higher prevalence of atopy.

On physical examination the tympanic membrane (eardrum) is observed from normal to atelectasic, with a yellow or blue colour due to the accumulation of fluid in the middle ear, turning to a brown colour when the process becomes chronic in nature. Usually the bright triangle of Politzer disappears when observing by otoscopy. Pneumatic otoscopy is the best diagnostic tool for OME because one can observe and identify the lack of movement of the tympanic membrane. Unfortunately, it is not widely used [15]. The diagnosis must be further supported by other investigations like audiometry and tympanometry. Audiometry will demonstrate a variable air bone-gap. Tympanometry will show a flat curve, because the tympanic membrane and the ossicular chain will not move due to the presence of fluid in the middle ear. There will be an absence of the acoustic reflex (Table 2).

**Table 2:** Symptoms and correlation with signs

Symptoms	Signs
Hearing loss	Conductive hearing loss
Autophony or fullness	Visible fluid behind the eardrum
Tinnitus	Dullness (Lost of Politzer bright triangle)

## Eosinophilic otitis media (EOM)

Eosinophilic otitis media (EOM) is an intractable form of otitis media characterized by the presence of a highly viscous yellow effusion containing eosinophils. EOM shows a very high rate of association with asthma. It is resistant to conventional treatments for otitis media. However, EOM associated with adult-onset asthma has been shown to improve following optimal asthma therapy [16, 17]. EOM predominantly affects women in their fifties.

High-tone hearing loss is more frequently found and more severe in EOM patients, and sudden deafness is also seen sometimes. EOM occurs bilaterally, mostly, although the onset of disease in each ear may differ. EOM is often associated with asthma both in non-atopic and atopic asthmatics. Studies looking at the relation of EOM and asthma severity have shown that asthma severity was statistically greater in patients with EOM than in patients without EOM. EOM is often complicated by eosinophilic rhinosinusitis [18, 19]. Moreover, there was a close relationship between EOM and asthma severity in asthma patients with chronic rhinosinusitis.

A significantly larger number of EG2- positive cells was observed in the middle ear mucosa of EOM patients than that of the control group (COM without bronchial asthma), proving that active eosinophilic inflammation is occurring in these patients [19]. Eosinophil chemoattractants like interleukin (IL)-5 and eotaxin, regulated on activation, normal T-cell expressed and secreted (RANTES); and ecalectin in middle ear effusion (MEE) are significantly higher in EOM patients than in controls [20, 21], not only at the protein level but also at the mRNA level, indicating that active eosinophilic inflammation occurs in the middle ear itself. The levels of ECP were found to correlate positively with that of IL-5, suggesting that IL-5 may play a crucial role in the accumulation of eosinophils in the middle ear [22]. Immunohistochemically, IL-5+ and ecalectin + cells are significantly increased in the middle ear mucosa of EOM patients as compared with that of the control group. Levels of IL-5 in MEE were significantly higher than those in blood in both groups of patients and in OME patients with asthma than in the control group. In addition, in OME patients with asthma, there was a significant correlation between the percentage of eosinophils and IL-5 levels in MEE [20]. Eotaxin levels in blood were significantly higher than those in MEE and eosinophilia in MEE depends more on IL-5 than on eotaxin, thus mobilizing eosinophils from the bone marrow into the blood.

In patients with EOM, 62% of patients (62%) versus 11% of control patients (11%) had antigen-specific IgE in the middle ear effusion although no difference was noted in the total serum IgE concentrations [23]. The severity score of EOM in the antigen-specific IgE-positive group was significantly higher than that in the antigen-specific IgE-negative group suggesting that antigen-specific IgE against inhalant and bacterial antigens may be locally produced in the middle ear mucosa in patients with EOM. In particular, local sensitization against fungi together with *Staphylococcus aureus* could result in local IgE production in the middle ear and may be responsible for the severity of EOM. The IgE positive cells were mainly mast cells, but also partially in the cytoplasm of cells that appeared to be plasma cells indicating the production of IgE locally in the middle ear mucosa. The existence of high-level IgE may exacerbate eosinophilic inflammation in the middle ear. The concentration of IgE in middle ear effusion significantly and positively correlated with bone conduction hearing levels at 2 kHz and 4 kHz in EOM patients. Overproduction of IgE locally in the middle ear may be related to the pathological condition of EOM and eventually cause inner ear damage.

Sixty-one percent of patients had extensive activation of mast cells in their middle ears [24]. Among those with elevated tryptase in their effusion, 95.6% were atopic and 94.7% also had elevated effusion eosinophilic cationic protein. Tryptase was elevated only in the effusion of atopic patients as compared with controls. Thus, in asthmatic patients who have a T-helper type 2–dominant predisposition, a patulous eustachian tube easily allows the entry of antigenic materials into the middle ear, causing an eosinophil-dominant inflammation. Eosinophils increase the production of mucin; and cytotoxic proteins derived from eosinophils impair the epithelial cells leading to a debris of infiltrating cells and epithelial cells mixed with excessively produced mucin, generating a more viscous effusion.

## Pathomechanisms and comorbidities of otitis media with effusion (OME)

The etiology of OME is believed to be multifactorial; with many different factors implicated in the pathophysiology of the disease [25–30]. Although dysfunction of the Eustachian tube (ET) is considered to be the main factor responsible for the development of OME [30], other host factors associated with the onset of OME include: upper respiratory tract infections (URTIs); mechanical obstruction of the nasopharynx by adenoid hypertrophy or craniofacial malformations such as cleft lip and palate [31] and Down’s syndrome [32, 33]; allergic and immunologic factors [34–36]; bacterial biofilms [37, 38]; and genetic factors [39, 40]. OME may occur as a consequence of acute otitis media (AOM) taking an extended period of time to resolve [41]. It is also now recognized that bacterial biofilms are crucial in the aetiopathogenesis of OME [37, 38, 42]. Confocal laser scanning microscopy has shown that bacterial biofilms are present as three-dimensional bacterial clusters encased in a self-produced amorphous extracellular matrix attached to a surface within the middle ear [38], and thought to exert a chronic inflammatory stimulus leading to OME. Similarly, recent guidelines from otologists, pediatricians, and allergists also support the role of allergy in the development of OME, based on clinical evidence [43–46]. Furthermore, there is some evidence that environmental and socioeconomic factors may also influence the pathogenesis of OME, such as: seasonal factors, history of allergy, the level of humidity, accessibility to health services, socioeconomic status, breast feeding duration, communal living environment, unhygienic habits, passive smoking and gastroesophageal reflux [47–52].

**Fig. 1:**
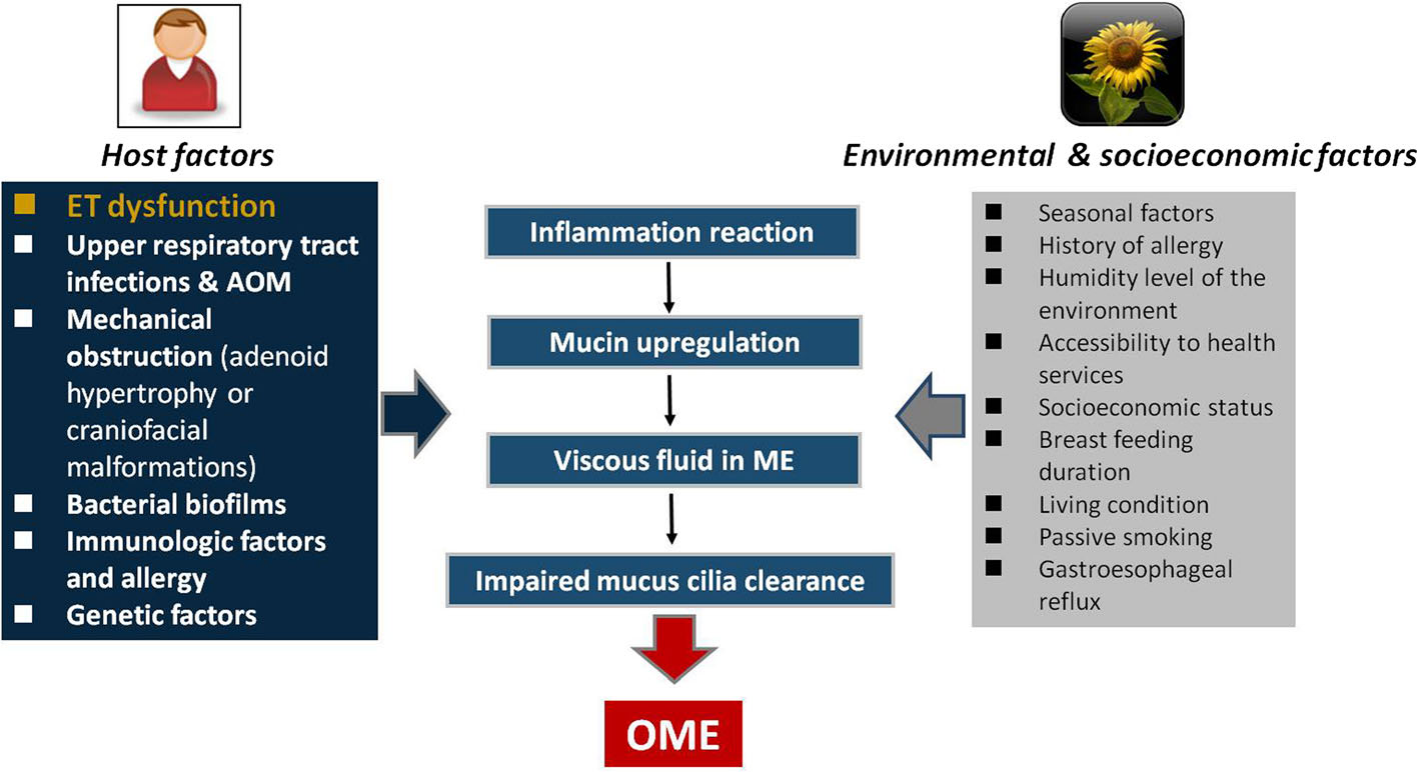
Pathogenesis of OME. Underlying factors. ET: eustachian tube; ME: middle ear; AOM: acute otitis media; OME: otitis media with effusion

While ET dysfunction has often been thought to cause middle ear effusion through negative pressure in the middle ear cleft, recent evidence suggests that ET dysfunction-associated middle ear effusion may be more complex as ET is considered to play a role in pressure regulation, secretion clearance, and protection from nasopharyngeal pathogens [53] (Fig. 1). Histologically, OME may be regarded as a chronic inflammatory condition, in which either a host or environmental/socioeconomic stimulus induces an inflammatory reaction in the middle ear mucosa [54] with an overproduction of mucin and production of altered more viscous mucin types [29], which overwhelms the normal mucociliary clearance system of the middle ear, resulting in functional blockage of the ET and subsequent accumulation of a thick, mucin-rich middle ear effusion [53].

When untreated, OME may lead to the development of adhesive otitis media, tympanic membrane abnormalities (e.g. chronic ear drainage), tympanic sclerosis, cholesterol granuloma, acquired primary cholesteatoma, other infections such as meningitis, and other sequelae [54–57]. Moreover, OME is associated with hearing loss and may cause permanent middle ear damage with mucosal changes. The hearing loss in OME is often transient as the middle ear effusion frequently resolves spontaneously, especially if OME follows an episode of AOM [58]. When OME is persistent, particularly if bilateral and early in life, it may impact negatively on speech development, education, and behavior of the affected individual; although the extent to which these features are affected can be variable and controversial [41]. Furthermore, persistent otorrhea and progressive hearing loss often result in a worsening quality of life of the affected individual.

## Role of allergy – IgE-mediated inflammation in the development of OME/EOM

The higher prevalence of otitis media with effusion (OME) in atopic children supported by the predominance of bilateralism and objective hearing loss suggests the important role of allergy in its genesis and recurrence. The prevalence of atopic conditions, including allergic rhinitis (AR) in patients with ROM, ranges from 24% to 89%. OME is often a co-morbidity of AR, while there are few reports on the role of non-allergic rhinitis in its causation [59, 60].

The allergy associated cytokines were assumed to act among the key regulators of the middle ear inflammation of chronic OME [61]. Eustachian tube functions can be affected directly by the mediators released in the nasal mucosa of patients with AR or indirectly by the resultant nasal obstruction [62]. In a multivariable analysis, AR, adenoiditis, and younger age were all shown to be independently associated with a diagnosis of OME [63]. A birth cohort of 291 children from Copenhagen in the 6th year of life revealed an association with AR (OR = 3.36, CI = 1.26–8.96, *P* = 0.02), but not with nasal mucosal swelling [64]. A significant association was reported between OME, eosinophil cationic protein values in middle ear effusion and persistent symptoms of AR [65].

The finding of marked elevation of effusion myeloperoxidase in atopic than non-atopic patients suggests that atopy may contribute to elevated levels of neutrophil activity in OME probably due to an enhanced response of the primed inflammatory cells to bacteria [66]. A recent investigation revealed that pro-inflammatory cytokines are found at high concentrations in middle ear effusion of both atopic and non-atopic children which may argue in favor of instituting anti-inflammatory management [67]. Trials of topical intranasal corticosteroids in the management of OME were shown to have promising initial results. However, a double-blind randomized placebo-controlled trial of intranasal corticosteroids in 4–11 year old children with persistent bilateral OME attending a primary health care setting revealed poor response [68].

OME occurring in the first 2 years of life was found to be associated with AR at 6 years of age as well as other allergic diseases [69, 70]. It was hypothesized that otitis media infections in early life, especially frequent or severe may influence the developing immune system, resulting in greater risk for developing late-onset allergic eczema and asthma during school age especially in those who had three or more attacks of OME [69, 71]. The likelihood of AR and asthma were found higher in patients with serous than with mucous effusion [72].

### Role of IgE mediated allergy in OME

IgE-mediated allergy has long been considered a causative factor in about one third of recurrent OME patients based on clinical observations and skin testing [73–75]. A meta-analysis of 24 studies revealed that the presence of atopy increased the risk of chronic and recurrent otitis media (OR, 1.36; 95% CI, 1.13–1.64; *p* = 0.001) [76]. Screening of 2320 children revealed a close association between atopy and the development of OME [77]. After multivariate analysis of the data taken from 88 one to seven year old children with OME, IgE sensitization, wheezing, nasal obstruction, family history of otitis, and child-care attendance were the main independent risk factors [78].

Atopy as indicated by skin prick test (SPT) results was evident in 11 out of 45 (24%) patients undergoing simultaneous tympanostomy tube placement for OME and adenoidectomy for adenoid hypertrophy [79]. In one investigation, 36.4% children with chronic or recurrent otitis media despite adenoidectomy and VT insertion had positive SPT for inhalant and food allergens. Allergy probably plays a more important role in recurrent rather than uncomplicated OME [80]. In another report, the incidence of atopy was 24% among 59 children with persistent OME [81]. SPT results were positive in 51/122 (41.8%) children with chronic OME participating in a randomized controlled trial. Twenty-two percent were positive to house dust mites (HDM), 13.9% to dog/cat allergens, 13.1% to Alternaria/Aspergillus mix, 10.7% to grass, 9.8% to cockroach, and 9% to ragweed [82].

The association with food allergy was also investigated in several studies. In one study, 78% of 104 unselected patients aged 1.5–9 years with recurrent OME were sensitized to one or more food allergens as revealed by SPT, specific IgE tests, and food challenges. An elimination diet of the suspected food resulted in the amelioration of OME in 86% of patients while reintroduction provoked recurrence in 94% (66/70) of patients over 16 weeks [83]. Recurrent OM has been reported in an extended follow-up of a cohort of 56 children with cow’s milk allergy as compared to 204 control children (27%, versus 12%, *p* = 0.009) [84]. Both chronic otitis media with effusion and Meniere’s disease were assumed to improve with treatment of food allergies [85].

### Role of IgE mediated allergy in EOM

Antigen-specific IgE against inhalant and bacterial antigens may be locally produced in the middle ear mucosa in patients with eosinophilic otitis media (EOM). One or more antigen-specific IgE antibodies were detected in middle ear effusion of 16 out of 26 patients (62%) with a higher severity score of EOM. Sensitization to fungi and *Staphylococcus aureus* in particular was observed [86].

Overproduction of IgE locally in the middle ear may be related to the pathological condition of EOM and eventually cause inner ear damage. The concentration of IgE in middle ear effusion significantly and positively correlated with bone conduction hearing levels at 2 kHz and 4 kHz in EOM patients [87].

EOM shows a very high rate of association with asthma [88]. After two months of omalizumab treatment, not only asthma, but also hearing loss improved in a case report [89].

## Similarities and differences between upper and lower airway inflammation in allergic rhinitis and rhinosinusitis and middle ear inflammation in OME/EOM

Clinical evidence supports the hypothesis that chronic OME is an allergic disease [44, 90, 91]. Furthermore, allergy is a unique comorbidity of OME and by far a greater risk factor than other identified contributing factors [36, 46, 92, 93]. Middle ear mucosa, which evolves from the same ectoderm as the rest of the upper respiratory tract epithelium, has been found in animal studies to have the same active intrinsic immunologic responsiveness to antigenic stimulus as the nasal tract, sinuses, and bronchi [94]. Indeed, it is now recognized that the upper respiratory tract responds as one unified airway and allergy can affect different target organs at different ages [95–97]. Nguyen and colleagues [98] have suggested that the middle ear may behave in a “similar manner to the lungs under allergic inflammatory insults” and that the “middle ear may be included in the united airways.” Furthermore, Parietti-Winkler and colleagues [99, 100] have suggested that the development of OME could be considered as a marker of severity of the inflammatory disease leading to nasal polyposis (NP), asthma and aspirin intolerance (AI), and that better characterization of NP patients with OME could allow to define more accurately the nature, type and severity of the underlying inflammatory process.

Although, the most frequently cited objection of past decades linking OME to the allergy hypothesis is that an allergen is unlikely to get into the middle ear itself because of the structural gatekeeper function of the ET [101], it has been hypothesized that secretory immunity does not rely solely on the premise of direct allergen transport to the middle ear but rather depends on both humoral and cell-mediated immunology [101]. Likewise, histological examination of middle ear effusion has demonstrated the presence of a large number of eosinophils, which are considered to be eosinophilic mucin. However, as not many eosinophils were observed in the middle ear mucosa as in the middle ear effusion, this lead to the speculation that eosinophils that migrated to the middle ear mucosa did not stay locally in the middle ear but migrated immediately to the middle ear cavity. In contrast, as the nasal and paranasal sinus mucosa of patients with AR and eosinophilic chronic rhinosinusitis show extensive accumulation of eosinophils in the submucosa, it is possible that the mechanism/s of eosinophil migration and survival may be different between middle ear mucosa and nasal mucosa of AR and NPs.

Importantly, however, immunoglobulin E (IgE) sensitization, a major contributing factor in multiple airway diseases including AR and NPs, has also been shown to independently increase the risk of OME regardless of mechanical obstruction [36]. This observation further supports the view that the middle ear may serve as a target organ for allergic reactions and that the consequences of atopy are related to defective reactions to external stimuli. Indeed, a recent study by Iino and colleagues [102] has demonstrated that EOM patients have a significantly higher level of IgE in middle ear effusion compared with control subjects. Furthermore, the IgE level in middle ear effusion was significantly higher than serum IgE level in the EOM patients, suggesting that IgE was likely to be produced locally in the middle ear mucosa [102]. Support for local IgE production comes from increasing recognition of local AR (LAR), a form of AR in a subgroup of idiopathic rhinitis individuals with negative allergy testing in whom inflammation is thought to be mediated by localized IgE, and allergen-specific IgE is also measurable in nasal secretions [103]. Similarly, Bachert and colleagues [104] have reported significant concentrations of IgE specific to *Staphylococcus aureus* enterotoxins in NP tissue and suggested that as the patients showed neither increase in serum antigen-specific IgE levels nor positive skin prick test reactions the *S. aureus* specific IgE was produced locally in the NPs (Fig. 1).

## Impact of upper airway or lower airway inflammation on OME/EOM. Is OME/EOM part of the chronic allergic respiratory syndrome?

Recent epidemiological studies support the evidence for the link between otitis media with effusion (OME) and allergy, especially allergic rhinitis (AR) [105, 106]. Roditi et al. used data of 1,491,045,375 pediatric visits from the National Ambulatory Medical Care Survey and National Hospital Ambulatory Medical Care Survey, 2005–2010. They found that age was an effect modifier of the association between AR and OME, and that a significant relationship was observed in children 6 years of age and older, whereas there was no significant association in younger children [105]. Kreiner-Møller et al. also analyzed data obtained from 291 children in the 6th year of life from the Copenhagen Prospective Studies on Asthma in Childhood (COPSAC) 2000 birth cohort. They reported that OME was diagnosed in 39% of the cohort and was significantly associated with AR, but not with nasal mucosal swelling, nasal eosinophilia, non-allergic rhinitis, asthma or eczema. The frequency of OME in British preschool children was reported from participants in the Avon Longitudinal Study of Pregnancy and Childhood (ALSPAC). Midgley et al. reported that there was a decrease in prevalence of OME with increasing age and a marked seasonal effect on the frequency of OME. It seems obvious that a link between AR and OME exists, but age effect is not yet clarified. Pathophysiological association between AR and OME has not been fully understood, but there were several clinical studies. Negative middle ear pressure following nasal allergen challenge in subjects with AR was reported 30 years ago and the involvement of both immunologic and mechanical mechanisms were hypothesized [107]. Gideon et al. tried to prove the pathophysiological mechanisms of OME as Th2 inflammation in the middle ear effusion of atopic individuals, but he hesitated to conclude that allergic inflammation was the cause of OME [108].

Since there may exist the pathophysiologic associations of AR with OME, treatments targeting allergic inflammation such as antihistamines, LTRA, intranasal corticosteroids or their combination might be useful in the management of OME, before considering surgical intervention [109].

## Conclusion

OME is highly prevalent in children. The hearing loss as a result of OME is a major health issue. Natural defense mechanisms of the upper airway via the epithelial defense barrier and innate immunity protects against environmental insults by microbes. Impairment of these defenses due to allergy may lead to increased susceptibility to infectious organisms in the respiratory tract and middle ear mucosa. In this context, it is known that Toll-like receptor signaling variations are associated with clinical phenotypes and the risk of infection in the middle ear. Allergy not only can induce an inflammatory reaction in the middle ear cavity but also increase susceptibility to infection by microbes. Although allergy plays a crucial role in the etiology of OME there are several other factors that play a role in the etiology of OME. One could hypothesize that as in asthma, there may be different phenotypes of OME, one of these, the most frequent is due to atopy.

### Unmet needs

1. Studies to assess precisely the pathogenesis of OME are needed.
2. Studies to analyze the relation between local environmental allergens and pollutants on OME/EOM in different geographic regions are needed.
3. Risk factors of OME is are know but studies on interventional strategies to reduce these risk factors and the impact on OME are needed.
4. Studies evaluating the impact of treatment of allergic rhinitis and rhinosinusitis on OME are needed.
